# DEGRO statement on the KEYNOTE-689 trial: Perioperative immunotherapy in combination with adjuvant (chemo-)radiotherapy for resectable locally advanced head and neck cancers

**DOI:** 10.1007/s00066-026-02511-6

**Published:** 2026-02-17

**Authors:** Sören Schnellhardt, Simon Böke, Silke Tribius, Mechthild Krause, Marlen Haderlein, Alexander Rühle, Christoph Süß, Annett Linge, Markus Hecht, Panagiotis Balermpas

**Affiliations:** 1 Department of Radiotherapy and Radiation Oncology, Saarland University Medical Center, Gebäude 6.5, Kirrberger Straße, 66421 Homburg, Germany; 2 Department of Radiation Oncology, University Hospital Tübingen, Eberhard Karls University Tübingen, Tübingen, Germany; 3 Department of Radiation Oncology, Asklepios Klinik St.Georg, Hamburg, Germany; 4 Department of Radiotherapy and Radiation Oncology, Faculty of Medicine and University, Hospital Carl Gustav Carus, TUD Dresden University of Technology, Dresden, Germany; 5 National Center for Tumor Diseases (NCT), NCT/UCC Dresden, a partnership between DKFZ, Faculty of Medicine and University Hospital Carl Gustav Carus, TUD Dresden University of Technology, and Helmholtz-Zentrum Dresden-Rossendorf (HZDR), Dresden, Germany; 6 Department of Radiation Oncology, Universitätsklinikum Erlangen, Friedrich-Alexander-Universität Erlangen-Nürnberg (FAU), Erlangen, Germany; 7 Department of Radiation Oncology, University Medical Center Leipzig, Leipzig, Germany; 8 Department of Radiotherapy, Hospital of the University of Regensburg, Regensburg, Germany; 9 Department of Radiation Oncology, University Hospital Zurich, University of Zurich, Zurich, Switzerland

The KEYNOTE-689 and NIVOPOSTOP studies are the first randomized phase III trials demonstrating a prognostic benefit of perioperative or adjuvant immune checkpoint inhibition (ICI) in the curative treatment of resectable locally advanced squamous cell carcinoma of the head and neck (LA-HNSCC) [1, 2].

The concept of perioperative immunotherapy—i.e., the combination of neoadjuvant and adjuvant immune checkpoint inhibition with tumor resection—has already been successfully established in other tumor entities such as early-stage non-small cell lung cancer, malignant melanoma, and triple-negative breast cancer [3–5]. It is assumed that immune activation in the perioperative setting and subsequent tumor resection have synergistic effects on the efficacy of adjuvant immunotherapy [6]. In phase II studies on LA-HNSCC, a similar concept demonstrated a prognostic advantage compared to historical control groups [7, 8].

The KEYNOTE-689 trial investigated the efficacy of the anti-PD1 ICI pembrolizumab as neoadjuvant (2 cycles pembrolizumab 200 mg, q3w) and adjuvant treatment (15 cycles pembrolizumab 200 mg, q3w) combined with current standard of care, consisting of surgery, followed by radiotherapy (RT) or in case of high-risk histology (positive resection margin of < 1 mm and/or extracapsular extension (ECE)) cisplatin-based chemoradiotherapy (CRT), compared to current standard of care in patients with untreated resectable LA-HNSCC (UICC stage III-IVa).

A total of 714 patients were randomized, most of whom had oral cavity cancer (61%) and laryngeal cancer (22%). Only a small proportion of patients with oropharyngeal cancer (10%) and hypopharyngeal cancer (8%) were included in the trial. Importantly, only 4% of patients in the trial had HPV-positive oropharyngeal cancer, indicating a marked underrepresentation of this subgroup. Patients with a PD-L1 CPS < 1 were also underrepresented (3.8%).

The study reached its primary endpoint, with the 3‑year event-free survival (EFS) significantly improved in the pembrolizumab group compared to the control group (total cohort: 3‑year EFS: 57.6% vs. 46.6%, hazard ratio (HR) 0.73, 95% confidence interval (CI) 0.58–0.92 *p* = 0.008). 3% of patients achieved a pathological complete response after 2 cycles of neoadjuvant pembrolizumab; a major pathological response was found in 9.3% of patients in the pembrolizumab group.

The demonstrated EFS benefit was mainly the result of an improvement in distant metastasis-free survival, while the risk of locoregional recurrence could not be reduced by perioperative immunotherapy compared to the control group.

In contrast, in the phase III NIVOPOSTOP trial conducted by GORTEC, only patients with high risk pathological features were randomized in the study (UICC stage III-IVb, presence of lymph nodes metastases with ECE, multiple perineural sheath invasion, ≥ 4 positive lymph nodes, R1 or ≤ 1 mm resection margin). Overall, 680 patients were randomized and received either standard of care treatment consisting of cisplatin-based CRT (cumulative dose 66 Gy, cisplatin 100 mg/m^2^ body surface area q3w) or standard therapy in combination with adjuvant nivolumab (one dose of nivolumab 240 mg before initiation of adjuvant CRT, 3 cycles of nivolumab 360 mg, q3w concurrent to CRT, and 6 cycles of nivolumab 480 mg, q4w as maintenance therapy). In this study, similar to KEYNOTE-689, the majority of treated patients had oral cavity cancers (58%) and only very few had HPV+ oropharyngeal carcinomas (5%). The primary endpoint of improved disease-free survival after 3 years was reached (63.1% vs. 52.5%, HR 0.76, CI 0.60–0.98, *p* = 0.034). In contrast to the KEYNOTE-689 trial, this study achieved an improvement in DFS through a reduction of locoregional recurrences rather than distant metastases.

The results of KEYNOTE-689 led to the FDA approval of pembrolizumab in June 2025 as neoadjuvant and adjuvant therapy for resectable LA-HNSCC in combination with RT or CRT for tumors with a CPS ≥ 1, and the NCCN guidelines have already been updated accordingly. EMA recently granted approval with an identical range of indications for Europe.

It is currently unclear whether adjuvant nivolumab therapy will also find its way into routine clinical practice.

## Recommendations for perioperative pembrolizumab therapy in clinical routine

1. PD-L1 CPS determination should be integrated into routine diagnostics for all resectable LA-HNSCC with immediate effect. Perioperative immunotherapy is only approved for tumors with CPS ≥ 1.
2. The decision on the use of perioperative immunotherapy should be made in an interdisciplinary setting. The decision on the primary treatment modality (resection + adjuvant therapy vs. definitive CRT) should be made independently of the use of perioperative ICI therapy. To undergo resection, patients should primarily be operable with good functional results. The effect of tumor reduction through neoadjuvant ICI therapy is small in most patients and cannot be compared to that of neoadjuvant chemotherapy. Furthermore, there are no data demonstrating the oncological safety of resection within the new borders, i.e. after tumor regression. Therefore, ICI is not to be used with the primary goal of improving operability of borderline resectable tumors.
3. The following applies to all locations: very advanced tumors in stage IVb (T4b and/or N3) were not included in the study population. If resection is possible in these cases, no evidence-based recommendation for or against perioperative ICI therapy can currently be made despite the high need for treatment.
4. Resectable locally advanced oral cavity squamous cell carcinomas should receive perioperative immunotherapy with pembrolizumab (Table 1).
5. Locally advanced HPV-positive oropharyngeal carcinomas should not receive perioperative immunotherapy. Although HPV-positive oropharyngeal carcinomas are included in the drug approval, they were considerably underrepresented in the KEYNOTE-689 trial. Moreover, patients with HPV+ locally advanced oropharyngeal carcinomas already have excellent loco-regional control rates of up to 100% after standard adjuvant (chemo-)radiotherapy [9]. Because of the very high response rates to standard therapy, de-escalation studies for HPV+ oropharyngeal carcinomas are ongoing (DELPHI trial (NCT03396718), [10]). Thus, any benefit from therapy escalation with ICI is unlikely. Adjuvant pembrolizumab showed no benefit in HPV-positive oropharyngeal carcinomas in the ADRISK phase II trial presented at ESMO 2025. Furthermore, in the CompARE phase III trial, durvalumab even led to reduced outcomes in HPV-positive oropharyngeal carcinoma treated with definitive CRT [11, 12]. Due to its very good efficacy, definitive chemoradiotherapy should always be offered as an alternative to resection to patients with HPV-positive oropharyngeal carcinoma.
6. Hypopharyngeal carcinomas and HPV-negative oropharyngeal carcinomas were underrepresented in the KEYNOTE-689 study, but could be considered for perioperative pembrolizumab therapy due to their unfavorable prognosis.
7. For hypopharyngeal and laryngeal carcinomas, the decision on whether surgical preservation of the larynx is feasible or not should also be made independently of the use of immunotherapy. If surgical resection is only possible by means of laryngectomy, larynx-preserving primary CRT (possibly with prior induction chemotherapy) should still be offered. The use of immunotherapies to improve the larynx preservation rate is subject of ongoing clinical trials [13]. In locally advanced tumors (T4aN0-2) and planned laryngectomy, the use of pembrolizumab is recommended.

**Table 1:** DEGRO recommendations for perioperative immunotherapy with pembrolizumab in resectable locally advanced squamous cell carcinomas of the head and neck (UICC stage III-IVa, 8th version TNM classification). All recommendations only apply to tumors with CPS ≥ 1. “+” = strong therapy recommendation, “+/−” = weak therapy recommendation, “−” = no therapy recommendation.

Tumor location	Clinical factors	Recommendation for perioperative pembrolizumab	Comments
*Oral cavity*	/	+	/
*Oropharynx*	HPV+	–	Consider definitive CRT as primary treatment
	HPV−	+/−	/
*Hypopharynx/larynx*	Primary larynx-preserving therapy appropriate and achievable (cT1‑3, N0-2)	–	Consider definitive CRT or induction chemotherapy + radiotherapy
	Not suitable for larynx-preserving CRT (cT4a, N0-2)	+	/
