# Correction to: DEGRO statement on the KEYNOTE-689 trial: Perioperative immunotherapy in combination with adjuvant (chemo-)radiotherapy for resectable locally advanced head and neck cancers

**DOI:** 10.1007/s00066-026-02534-z

**Published:** 2026-04-24

**Authors:** Sören Schnellhardt, Simon Böke, Silke Tribius, Mechthild Krause, Marlen Haderlein, Alexander Rühle, Christoph Süß, Annett Linge, Markus Hecht, Panagiotis Balermpas

**Affiliations:** 1 Department of Radiotherapy and Radiation Oncology, Saarland University Medical Center, Gebäude 6.5, Kirrberger Straße, 66421 Homburg, Germany; 2 Department of Radiation Oncology, University Hospital Tübingen, Eberhard Karls University Tübingen, Tübingen, Germany; 3 Department of Radiation Oncology, Asklepios Klinik St.Georg, Hamburg, Germany; 4 Department of Radiotherapy and Radiation Oncology, Faculty of Medicine and University, Hospital Carl Gustav Carus, TUD Dresden University of Technology, Dresden, Germany; 5 National Center for Tumor Diseases (NCT), NCT/UCC Dresden, a partnership between DKFZ, Faculty of Medicine and University Hospital Carl Gustav Carus, TUD Dresden University of Technology, and Helmholtz-Zentrum Dresden-Rossendorf (HZDR), Dresden, Germany; 6 Department of Radiation Oncology, Universitätsklinikum Erlangen, Friedrich-Alexander-Universität Erlangen-Nürnberg (FAU), Erlangen, Germany; 7 Department of Radiation Oncology, University Medical Center Leipzig, Leipzig, Germany; 8 Department of Radiotherapy, Hospital of the University of Regensburg, Regensburg, Germany; 9 Department of Radiation Oncology, University Hospital Zurich, University of Zurich, Zurich, Switzerland

**Correction to:**

**Strahlenther Onkol 2026**

10.1007/s00066-026-02511-6

In this article, the Conflict of interest statement for Simon Böke was incorrectly given as ‘no competing interests’ but should have been ‘S. Böke: Merck (speaker fees, travel expenses), Elekta (speaker fees, travel expenses), Regeneron (speaker fees).’

The original article has been corrected.

